# Supplementary material for: An atlas connecting shared genetic architecture of human diseases and molecular phenotypes provides insight into COVID-19 susceptibility
Source: Genome Med. 2021 May 17;13:83. doi: 10.1186/s13073-021-00904-z (PMC8127495; doi:10.1186/s13073-021-00904-z)
Supplement: Supplementary file 2 — Additional file 2: Figure S1. Clumping of GWAS results from NHGRI-EBI GWAS catalog. [file 13073_2021_904_MOESM2_ESM.pdf]

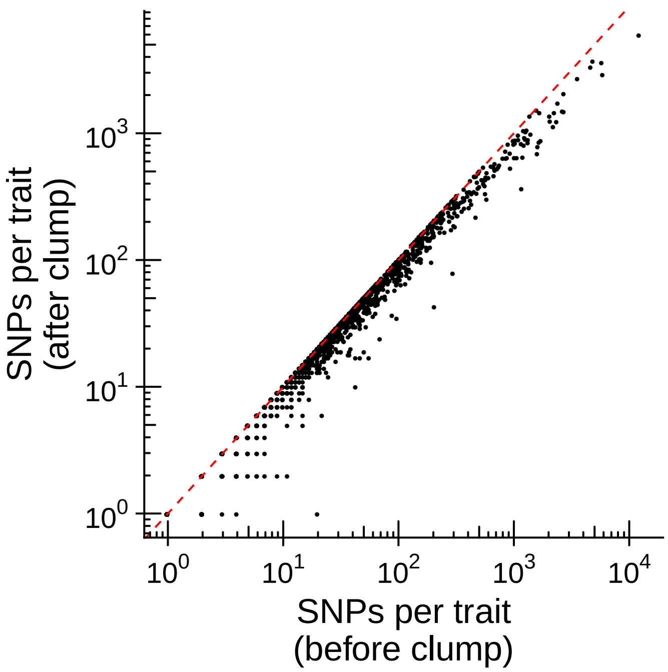

Fig. S1. Clumping of GWAS results from NHGRI-EBI GWAS catalog. For each GWAS traits, we performed LD clumping to only keep the lead SNP in each region.
