# Supplementary material for: An atlas connecting shared genetic architecture of human diseases and molecular phenotypes provides insight into COVID-19 susceptibility
Source: Genome Med. 2021 May 17;13:83. doi: 10.1186/s13073-021-00904-z (PMC8127495; doi:10.1186/s13073-021-00904-z)
Supplement: Supplementary file 4 — Additional file 4: Figure S2. Histogram of shared SNPs for each trait pair in NHGRI-EBI GWAS catalog from iCPAGdb at false discovery rate of 0.1. [file 13073_2021_904_MOESM4_ESM.pdf]

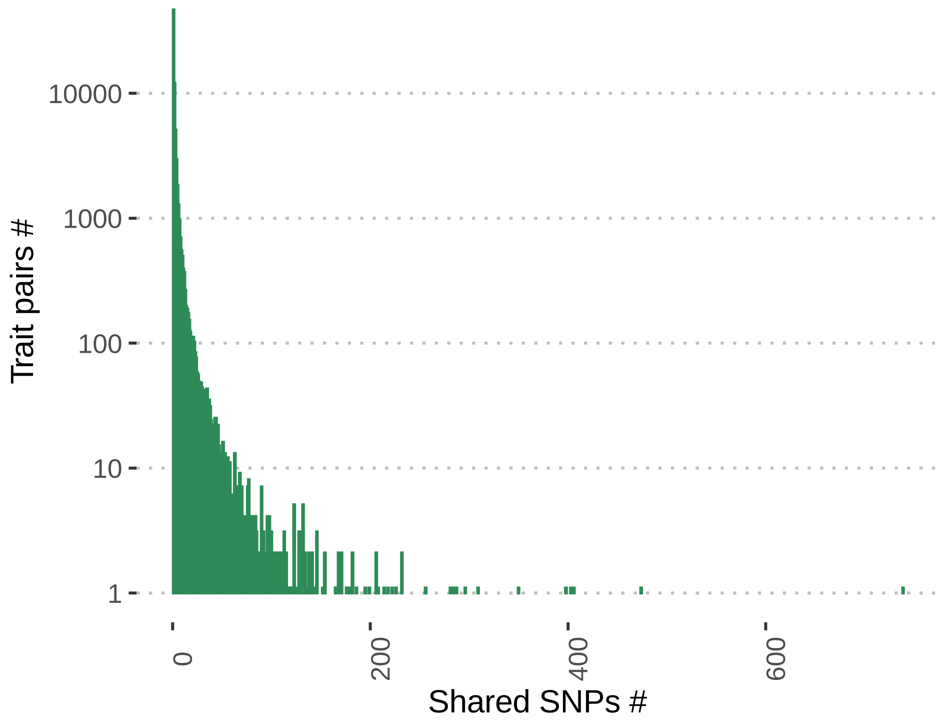

Fig. S2. Histogram of shared SNPs for each trait pair in NHGRI-EBI GWAS catalog from iCPAGdb at false discovery rate of 0.1. Among 76127 trait pairs (including compound phenotypes), the mean number of shared independent SNPs was 3.10, and 38.8% of trait pairs shared more than 1 SNP. “Mathematical ability” and “self reported educational attainment” shared the most SNPs with 739.
