# Supplementary material for: An atlas connecting shared genetic architecture of human diseases and molecular phenotypes provides insight into COVID-19 susceptibility
Source: Genome Med. 2021 May 17;13:83. doi: 10.1186/s13073-021-00904-z (PMC8127495; doi:10.1186/s13073-021-00904-z)
Supplement: Supplementary file 5 — Additional file 5: Figure S3. Comparison of CPAG1 and iCPAGdb using the NHGRI-EBI GWAS catalog summary statistics downloaded on September 4, 2013. [file 13073_2021_904_MOESM5_ESM.pdf]

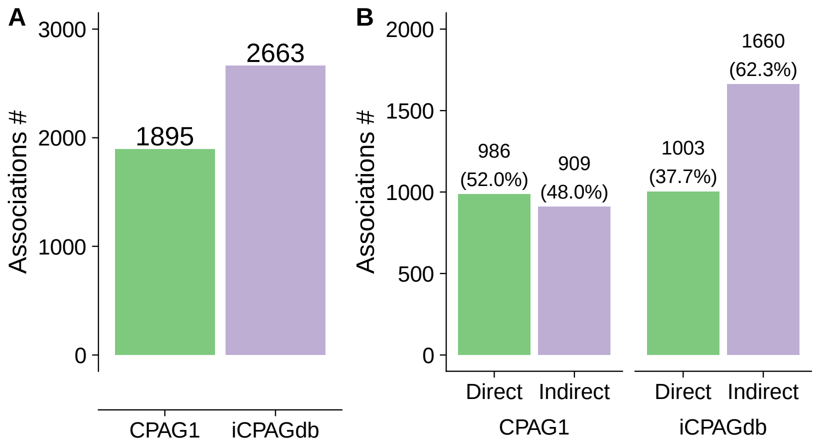

Fig. S3. Comparison of CPAG1 and iCPAGdb using the NHGRI-EBI GWAS catalog summary statistics downloaded on September 4, 2013. CPAG1 finished this analysis in 73.2 minutes while iCPAGdb used 4.1 minutes on a MacBook with Quad-Core intel i7 CPU and 16 GBs of RAM. CPAG1 cannot run efficiently enough to allow for comparison with the 2020 NHGRI-EBI GWAS catalog (estimated completion time > ~10 days for CPAG vs. ~100 minutes for iCPAGdb). A total of 887 traits were included in the dataset, and p values were corrected using multiple test correction for 392,941 comparisons. A) iCPAGdb detected 2598 cross-phenotype associations, 37% more than CPAG1 at FDR of 0.1. B) The number of cross-phenotype associations from directly shared SNPs were nearly unchanged between iCPAGdb and CPAG1 but indirect associations increased 76%.
